# Analysis of Relative Scent Intensity, Volatile Compounds and Gene Expression in *Freesia* “Shiny Gold”

**DOI:** 10.3390/plants9111597

**Published:** 2020-11-17

**Authors:** Aparna Srinivasan, Myung Suk Ahn, Gyeong Suk Jo, Jung Nam Suh, Kyung Hye Seo, Won Hee Kim, Yun Im Kang, Young Ran Lee, Youn Jung Choi

**Affiliations:** 1 Floriculture Research Division, National Institute of Horticultural and Herbal Science, Rural Development Administration, Wanju 55365, Korea; aparna3@outlook.com (A.S.); ahnms@korea.kr (M.S.A.); suhjn@korea.kr (J.N.S.); seokh@korea.kr (K.H.S.); rosewh@korea.kr (W.H.K.); yunimy@korea.kr (Y.I.K.); leeyr628@korea.kr (Y.R.L.); 2 Environment-Friendly Agricultural Reasearch Institute, Jeollanamdo Agricultural Research and Extension Service, Najusi, Jeollanamdo 58213, Korea; hyeong21@kore.kr

**Keywords:** *Freesia hybrida*, floral scent, volatile terpenes, GC–MS, *TPS*, linalool synthase

## Abstract

Scent is one of the most important economic traits in *Freesia hybrida*. “Shiny Gold”, a popular cultivar in South Korea, is widely cultivated for its scent. The relative scent intensity of “Shiny Gold” was approximately 16% higher in full-bloomed flower when compared to the yellow bud stage, while tissue-specifically, tepals showed higher intensity in electronic-nose (e-nose) analysis. E-nose analysis also showed that the scent intensity of “Shiny Gold” was higher and lower than “10C3-424” and “10C3-894”, respectively, and was similar to “Yvonne”. These results correlated to those of the olfactory tests. In total, 19 volatile compounds, including linalool, β-ocimene, D-limonene, trans-β-ionone were detected in gas chromatography–mass spectrometry analysis. Among these, linalool was the major volatile compound, accounting for 38.7% in “Shiny Gold”. Linalool synthase and *TPS* gene expression corresponded to the scent intensity of the four cultivars, with the lowest expression in the “10C3-424”. *TPS 2*, *TPS 3*, *TPS 5*, *TPS 6* and *TPS 8* were highly expressed in both bud and flower in “Shiny Gold”, while the expression of *TPS 4* was lower, relative to other *TPS* genes in both the flowering stages. These results may aid in enhancing scent composition in *Freesia* cultivars using marker-assisted selection.

## 1. Introduction

*Freesia hybrida* originated in southern Africa, belongs to the family of Iridaceae and is widely cultivated across the world. It is majorly grown for cut flower and potted plant production. Cultivation of *Freesia* has increased as they can thrive even at low temperatures of about 8–23 °C during winter, which reduces greenhouse production expenses by lowering heating costs. They have attractive flowers with diverse colors, flower shape and is known for its sweet scent. The floral scent of *Freesia* helps in attracting pollinators and also functions as a defense mechanism against microorganisms [1,2,3]. It also triggers a sense of happiness and comfort in humans [4], which has led to increased interest in floral scent research.

During the 1890′s *Freesia*, breeding involved diploid species such as *F. alba*, *F. refracta*, *F. corymbosa* and *F. leichtlinii*, which had a sweet scent. In the 20th century, various strains were bred, including *F. tubergenii*, *F. chapmanii* and *F. ragionieri*. Through interspecific hybridization, the first tetraploid *Freesia* “Butter Cup” was developed [5]. Tetraploid cultivars are characterized by a bigger sized flower with diverse colors. A few *Freesia* cultivars have lost scent due to breeding emphasis on visual esthetics and postharvest vase life [6]. However, as consumers prefer scented cultivars, breeding objectives in *Freesia* must also include selection for scent traits. In South Korea, the value of cut flower and pot plant production accounts for 87% and 12%, respectively, while potted plant production for indoors in the winter season is growing approximately over 10 times for the past ten years [7]. The scent has become more important in *Freesia*, and its scope of breeding has increased.

Scent characterization can be performed through an olfactory test, but it requires a trained panel and limits reproducible results. To overcome the disadvantage of olfactory tests, sensory evaluations of *Freesia* cultivars have been performed using untrained respondents with seven-point bipolar and five-point monopolar scales and the data are then analyzed using principal component analysis [8]. Volatile compounds are identified using gas chromatography–mass spectrometry (GC–MS) analysis, and this instrument determines specific compounds; however, they cannot provide fingerprint data on the overall fragrance pattern. Another instrumental method adopted for scent profiling is E-Nose, which has been used to characterize scent pattern and relative scent intensity in ornamental plants like Cymbidium [9], *Amorphophallus titanum* [10] and also *Freesia* [11]. Studies have focused on molecular mechanisms underlying scent production in other ornamental crops like *Polianthes tuberosa* [12], *Osmanthes fragrans* [13] and *Lilium* [14]. In *Freesia*, using GC–MS analysis, studies have reported that linalool, α-terpineol and ionones are the major volatile compounds [15], while terpene synthase (*TPS*) genes play an important role in volatile compound biosynthetic pathways [16,17].

In this study, the floral scents of four cultivars were comparatively analyzed to check whether their scent pattern, the relative content of volatile compounds, and gene expression corresponds to the emission of strong or weak scent. The following analyses were performed; (i) the relative scent intensity of different tissues and developmental stages, including postharvest storage, were measured using e-nose sensors. (ii) the volatile compounds responsible for scent were identified using HS-SPME–GC–MS analysis. (iii) Moreover, finally, the relative expression of *TPS* and linalool synthase genes were quantified. The comparative study could be useful for breeding scented *Freesia* cultivars, which may be used as resource material in cosmetic and perfume industries.

## 2. Results and Discussion

### 2.1. Relative Scent Intensity in “Shiny Gold”

**Figure 1:**
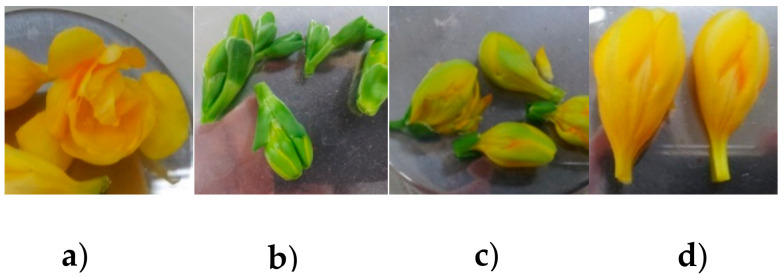
Floral developmental stages of “Shiny Gold”; (**a**) green, (**b**) green-yellow, (**c**) yellow and (**d**) full-bloomed.

**Figure 2:**
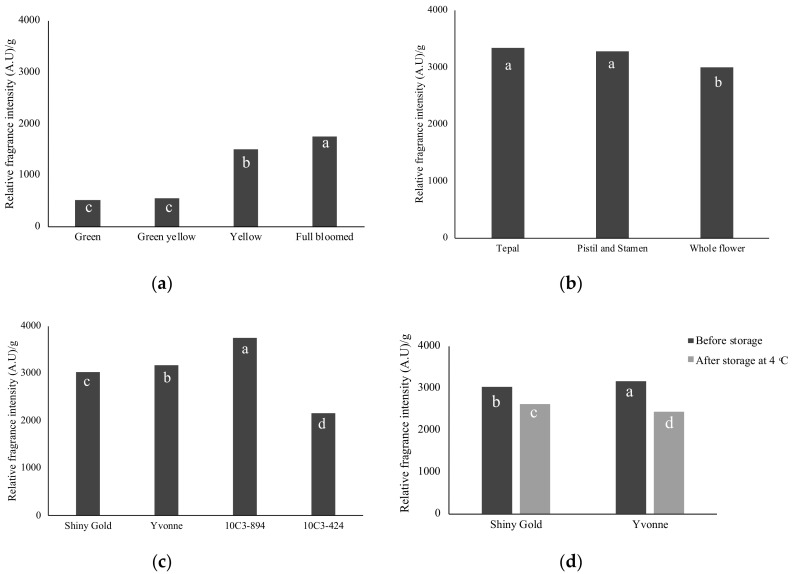
Relative scent intensity using mobile e-nose sensor in (**a**) developmental stages of “Shiny Gold”; (**b**) floral tissues of “Shiny Gold”; (**c**) four *Freesia* cultivars and (**d**) “Shiny Gold” and “Yvonne” after storage at 4 °C. Different letters indicate significant differences among means according to ANOVA analysis (*p* < 0.05).

The olfactory test was performed by a trained panel of eight individuals. The results showed that the floral scent of ‘10C3-894’ was evaluated as very strong (level 5-very strong) followed by “Shiny Gold” and “Yvonne” with the same level of fragrance (level 4-strong) (Table 1). Mobile e-nose was used to analyze scent emission in different tissues and stages of *Freesia* “Shiny Gold” (Figure 1). The scent intensity of entire flower stages, including green bud, yellow bud, initial open flowering and fully opened, showed 511, 555, 1505 and 1746 A.U (Figure 2a), respectively, the intensity of scent emission, gradually increased just before flowering. The scent was collected for 6 h from petal, sexual organs and entire flower of “Shiny Gold”; the intensity showed 3347 A.U, 3279 A.U and 3035 A.U, respectively (Figure 2b). The floral scent intensity of “Yvonne” was 3,166.5 A.U, which was 131.5 A.U, higher than “Shiny Gold”. The floral scent intensities of “10C3-894” and “10C3-424” were 3749 A.U and 2165 A.U, corresponding to strong and less-strong scent characteristics in the field (Figure 2c). Scent increases from bud to full flowering stage to aid in attracting pollinators, and the intensity decreases post-flowering [18]. Our results showed that floral parts, including tepal, stamen and pistil, emit scent in *Freesia*, which concurs with a previous observation [17]. From the e-nose result, “Shiny Gold” and “Yvonne” had relatively equal fresh flower scent intensity. Cut stems after harvest require a duration of approximately one week from farm to consumer. Therefore, the postharvest scent retention was examined in cut flowers of “Shiny Gold” and “Yvonne” after storing for seven days at 4 °C (Figure 2d). The result showed that relative scent intensity was higher in “Shiny Gold” (2620 A.U) when compared with “Yvonne” (2441 A.U), indicating that “Shiny Gold” retains scent, post storage.

### 2.2. Comparison of Relative Scent Intensity in Freesia Cultivars

**Figure 3:**
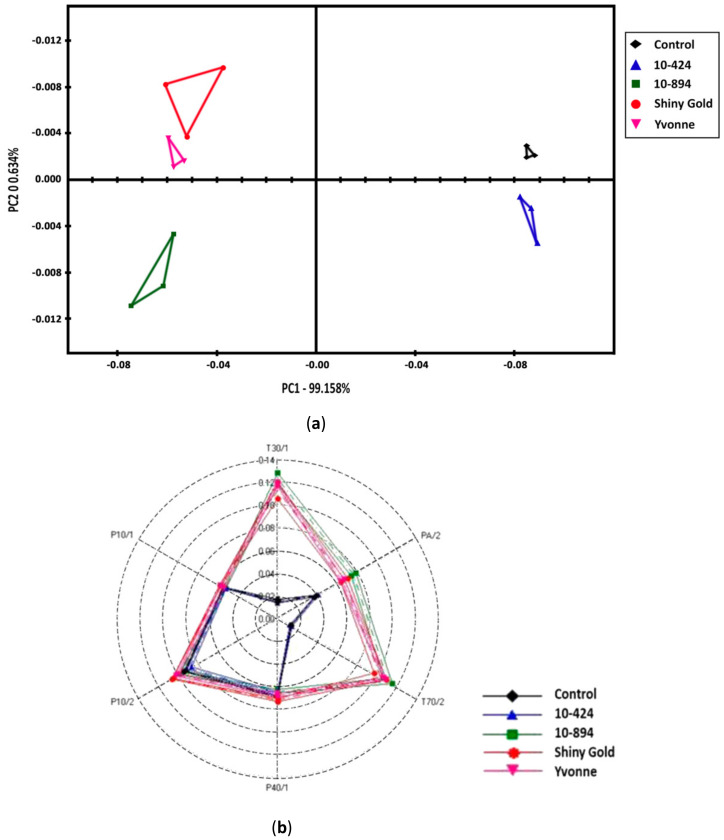

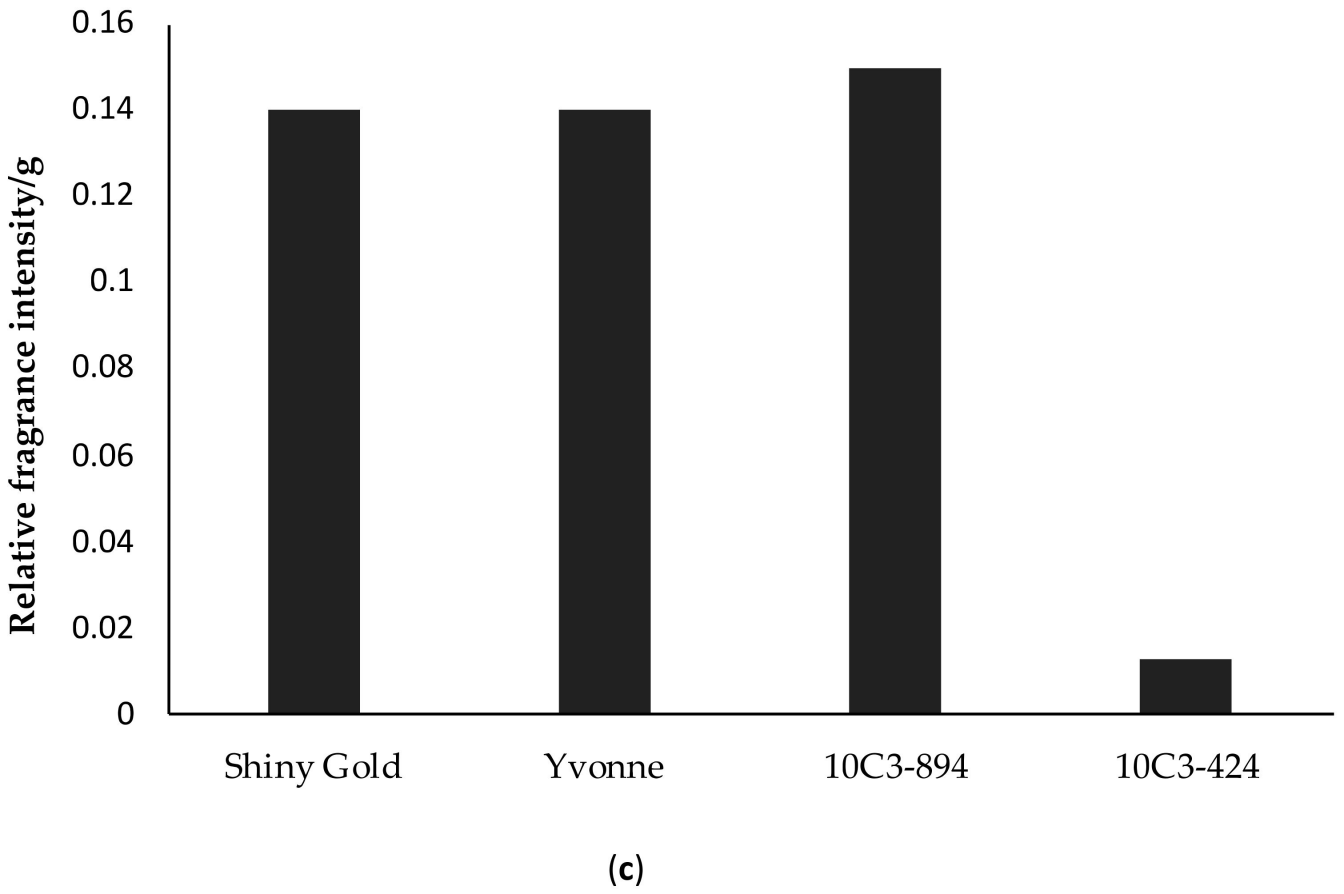
Relative scent analysis in four *Freesia* cultivars using an e-nose sensor. (**a**) PCA, (**b**) radar plot, (**c**) relative scent intensity (sensor response).

Relative scent intensity was measured and validated using two e-nose sensors (e-nose, Alpha MOS and mobile e-nose, FATUBA). Principal component analysis using e-nose (Alpha MOS) showed that (Figure 3a), PC1 and PC2 captured the largest percentage of variation. The first principal component, PC1, represents 99.2% and PC2, represents 0.6% of the total variance (99.8%). The relative intensity is the measure of Euclidean distance between the centroid of control and samples. In Figure 3a, the three replicates of control clustered into a small group, which indicates the uniformity of samples without variation. From Figure 3b, the response intensity was lowest in 10C3-424, which was similar to control (air). Therefore, we could assume that the “10C3-424” cultivar has a weaker scent compared to other cultivars. Further, the small clusters formed from replicates indicate the reliability and reproducibility of the tests. In the PCA plot, we could see that each cultivar had clustered at different quadrants, which may be indicative of each cultivar’s unique scent pattern. As expected, scented cultivars clustered distantly from control, whereas the weakly scented “10C3-424” was found in close proximity with the control. In addition, PCA categorizes samples based on relative intensity without considering the amount of volatile compounds [19]. Earlier studies have shown that e-nose sensors clusters unscented or less scented near control [20]. The radar plot showed significant variation in pattern between the cultivars. In the radar plot among the six sensors, four sensors, namely T30/1, PA/2, T70/2 and P10/2, showed a higher response (Figure 3b). Combining the response outlined above, these sensors show their greater affinity towards organic solvent, volatile compound, fluoride/chloride and lesser response for nonvolatile compounds. The sensor data (Figure 3c) denoted that “Shiny Gold” and “Yvonne” had the same relative intensity (0.14), “10C3-894” had the highest relative intensity (0.15), whereas “10C3-424” showed the least relative intensity (0.01).

### 2.3. Volatile Compounds of Four Freesia Cultivars Detected by HS-SPME-GC–MS Analysis

HS-SPME combined with GC–MS analysis revealed a total of 19 volatile compounds in the four different *Freesia* cultivars, namely “Shiny Gold”, “Yvonne”, “10C3-894” and “10C3-424” (Table 2). Among the 19 volatile compounds, 15 compounds were monoterpenes while four were sesquiterpenes (α-cubebene, α-cyperene, trans-β-ionone and α-selinene). Sixteen volatile components were identified in the volatile profile of “Shiny Gold”, accounting for 89.6 % of total volatile content. It is noticeable that “10C3-424” has a poorer profile of compounds with a relative content of 28.4%. The predominant volatile compounds in “Shiny Gold” were 2-norpinene,3,6,6-trimethyl (2.4%), β-myrcene (3.6%), D-limonene (5.0%), β-ocimene (29.6%), linalool (38.7%), allo-ocimene (1.3%), 2,4,6-octatriene,2,6-dimethyl-,(E,E)- (1.8%) and α-terpineol (4.5%). In ‘10C3–894′, the major compounds found were β-myrcene (5.8%), D-limonene (5.2%), 2-norpinene, 3, 6, 6-trimethyl (1.5 %), β-ocimene (3.4%), linalool (62.4%), α-terpineol (4.1%), α-cubebene (2.4%) and α-selinene (2%). In “Yvonne”, β-myrcene (4.1%), 2-norpinene,3,6,6-trimethyl (2.6 %), β-ocimene (27.1 %), terpinolene (1.0%), linalool (40.9%), 2,4,6-octatriene,2,6-dimethyl-,(E,E)- (3.2%), allo-ocimene (2.1%) and α-terpineol (1.7%) were the major compounds while in “10C3-424”, only D-limonene (3.5%), β-ocimene (1.5 %) and linalool (23.4%) were detected. Although linalool was found in all the cultivars, the highest level was measured in “10C3–894” (62.4%). Linalool synthase is the key enzyme, which catalyzes the formation of linalool. Previous studies have shown that linalool is among the major floral volatile compounds emitted not only in *Freesia* [21] but also in other flowering plants such as *Clarkia breweri* [22]. Besides their role in aiding pollination, few studies speculate linalool may also have a defensive response against biotic stress [23]. β-ocimene was the second major compound in “Shiny Gold” and “Yvonne”, and its role in pollination has been reported in oriental lilies [24]. Previous studies have detected volatile compounds in *Freesia* cultivars, including “Red River” and “Ambiance”. In “Red River”, the predominant compounds were monoterpenes such as α-pinene, β-pinene, 1,8- cineole, D-limonene, cis-ocimene, terpinolene and α-terpineol, while most of the compounds in “Ambiance” were sesquiterpenes, namely hotrienol, copaene, elemene, α-gurjunene, caryophyllene, α-guaiene, α-patchoulene, ϒ-cadinene, selinene and vatirenene [17]. This shows that the relative proportion of volatile compounds varies among the *Freesia* cultivars, and there is a great diversity of volatiles between them. Moreover, scent related studies have indicated that the scent pattern may vary within species or cultivars [25]. In conclusion, terpenoid biosynthesis generates diverse scent profiles among *Freesia* cultivars.

### 2.4. Gene Expression in Flowering Stages

*TPS* genes were found to play a predominant role in scent production in flower tissues of *Freesia hybrida* cultivars [17]. *TPS* genes catalyze multiple products from a single substrate by cyclization, termination reaction and loss of protons resulting in diverse volatile terpenes [26,27,28]. Studies have characterized the function of various *TPS* genes; α-terpineol is catalyzed by *TPS 3*, while volatile compounds such as linalool, α-terpineol, 1,8-cineole, D-limonene, 4-terpineol, myrcene and α-pinene are catalyzed by *TPS 2*. *TPS 6* catalyzes the formation of myrcene, ocimene, terpinolene, while *TPS 8* catalyzes the formation of monoterpenes and sesquiterpenes [16,17,27]. *TPS 4* was responsible for the synthesis of linalool, especially in calyx and torus [17]. The biochemical characterization of *TPS 5* revealed its inability to produce sesquiterpenes [17].

#### 2.4.1. TPS Expression in “Shiny Gold”

**Figure 4:**
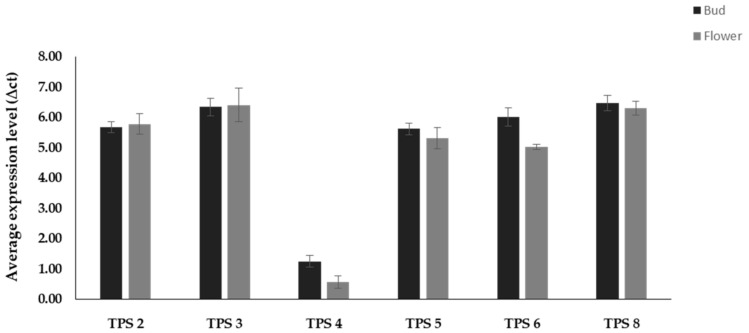
Average expression of *TPS* genes in “Shiny Gold” during flowering stages. *Y*-axis shows the average expression level (∆ct) in gene expression relative to the reference gene (ASK1). Bars represent mean ± SD, *n* = 3.

In the present study, qRT-PCR analysis showed that the *TPS* gene is expressed in both bud and full-bloomed stages in “Shiny Gold” (Figure 4). Though scent intensity due to volatile compound emission was found to be highest in the full-bloomed stage through e-nose based sensorial analysis, the expression of *TPS* genes in both bud and full-bloomed stage may be due to the role of *TPS* genes in carotenoid production and plant hormone biosynthetic pathways, other than in the biosynthesis of volatile compounds [29]. Among the six *TPS* genes, *TPS 4* expression was relatively lower; this may be due to the use of tepals instead of calyx or torus tissues in the q-RT-PCR analysis [17]. The strong scent observed in “Shiny Gold” may have been due to the catalysis of diverse monoterpenes by *TPS* genes.

#### 2.4.2. Relative Expression of TPS Genes in “Shiny Gold” in Comparison to Three Cultivars

**Figure 5:**
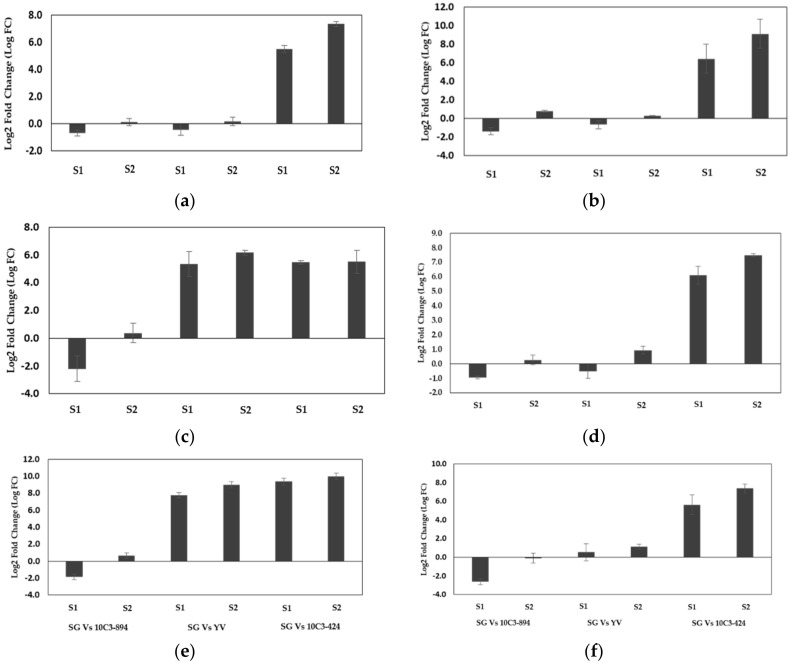
Comparative terpene synthase *(TPS)* gene expression among *Freesia* cultivars. (**a**) *TPS 2*, (**b**) *TPS 3*, (**c**) *TPS 4*, (**d**) *TPS 5*, (**e**) *TPS 6*, (**f**) *TPS 8*. *Y*-axis shows log _2_ (fold change) in gene expression relative to the reference gene (ASK 1). Bars represent mean ± SD, *n* = 3.

The expression of *TPS* genes in “Shiny Gold” was compared with “Yvonne”, “10C3-894”, and “10C3-424”. The results are presented in Figure 5. Between “Shiny Gold” and “Yvonne”, *TPS 4* showed an approximately 5.3-fold and 6.2-fold increase in stages; S1 and S2, respectively, while *TPS 6* was highly expressed approximately by 7.8 and 9.0-fold in S1 and S2, respectively. Three genes, namely *TPS 2*, *TPS 3* and *TPS 5*, were down-regulated in S1. The differences in volatile profiles between “Shiny Gold” and “Yvonne” might be associated with *TPS 4* and *TPS 6* expression. Moreover, *TPS 4* and *TPS 6* could regulate the formation of monoterpenes like linalool, myrcene, limonene and ocimene. Further, between “Shiny Gold” and ‘10C3–894′, all the six *TPS* genes were down-regulated in S1, whereas in S2, there was no significant change in their expression level. Though there was not much change in expression level, variation in components of one or two volatile compounds might have resulted in differences in scent emission. In the terpene biosynthetic pathway, α-terpineol, limonene and 3-carene belong to similar biosynthetic routes [27]. This is one of the reasons for the distinct floral scent profile in different species or varieties [17]. In “10C3-424”, all the six *TPS* genes were upregulated in both S1 and S2. As expected, the weak cultivar had no to very low expression of the six *TPS* genes. The expression of all the genes was relatively higher in the full-bloomed stage. Among the six genes, *TPS 6* and *TPS 2* exhibited higher expression. *TPS 6* was nearly 9.4-fold higher in S1 and 10.0-fold higher in S2, whereas *TPS 2* was about 6.4 and 9.0-fold higher in S1 and S2, respectively. Previous studies have reported that the relative gene expression is higher at the full-bloomed stage and reduces post-flowering [18]. Overall, there was a clear difference in the expression levels of *TPS* genes between strong and weak scented cultivars. Studies in *Alstroemeria* [30] and *Lillium* [31] have shown that increased scent emission results from high expression of *TPS* genes in scented cultivars. From our study, we could infer that differences in the level of *TPS* expression may alter the scent profile and floral scent intensity between cultivars.

#### 2.4.3. Linalool Synthase Gene Expression in Four Cultivars

**Figure 6:**
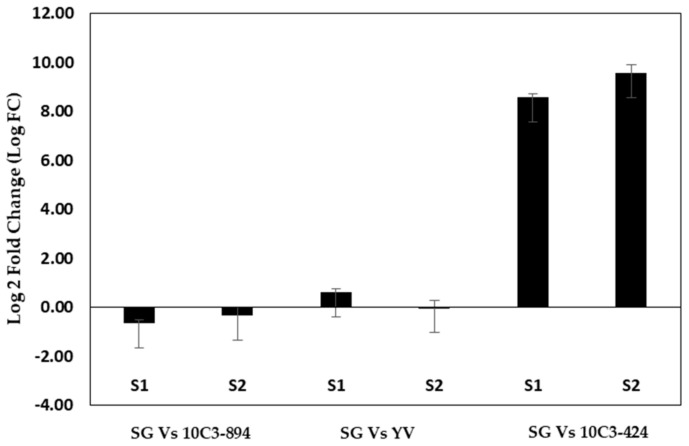
Linalool synthase gene expression among *Freesia* cultivars. *Y*-axis shows log _2_ (fold change) in gene expression relative to the reference gene (ASK1). Bars represent mean ± SD, *n* = 3.

Studies in *Freesia* have shown linalool as the major volatile compound [15,16,32,33]. Linalool synthase (LIS) catalyze linalool [22]; therefore, LIS gene expression was checked in strong and weak scented cultivars (Figure 6). As expected, the results showed the highest expression in “10C3–894”, followed by “Shiny Gold” and “Yvonne”, which were strong-scented cultivars. “Shiny Gold” showed higher expression in S1, whereas, in “Yvonne”, S2 showed higher expression. In the weak scented cultivar (“10C3-424”), the expression of the *LIS* gene was downregulated in both S1 and S2. Previous studies have shown that though *Clarkia breweri* and *Clarkia concinna* have a functional linalool synthase gene, only *Clarkia breweri* emits linalool. The difference between linalool emitter and non-emitter is suggested to be due to the differences in the regulation of *LIS* gene expression [22]. Our results concur with this. Our study showed that linalool is a major volatile compound in *Freesia* and is controlled developmentally. Further, these results may be useful for selecting ideal parents for breeding and manipulating volatile profiles, which may enhance the commercial value of *Freesia*. Moreover, *Freesia* emerges in diverse colors, wherein certain pink and purple colored cultivars are considered attractive, but they lack scent [5]. Manipulating *TPS* gene expression with recombinant DNA technology could be exploited for inducing scent in such cultivars.

## 3. Materials and Methods

### 3.1. Plant Materials

**Figure 7:**
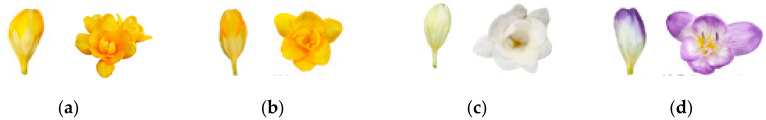
Bud (S1) and full-bloomed (S2) stages of cultivars used in the study; (**a**) “Shiny Gold”, (**b**) “Yvonne”, (**c**)’10C3-894′ and (**d**) “10C3-424”.

*Freesia* cultivars were grown in a glasshouse maintained at 8–25 °C in NIHHS, RDA, South Korea. Four cultivars were used in the study, namely, “Shiny Gold”, “Yvonne”, “10C3-894”, and “10C3-424” (Figure 7). They were selected based on the following criteria: “Yvonne” was selected as it was cultivated widely in Netherland and also possesses a strong scent like “Shiny Gold”. “10C3-894” was included for a very strong scent while “10C3-424” for weak scent.

### 3.2. Floral Samples for E-Nose Analyses

Two e-nose sensors (Alpha MOS FOX-2000 and mobile e-nose, FUTABA, Mobara, Japan) were used to measure the relative scent intensity in *Freesia* cultivars. Different tissues and stages were used in the study. For e-nose analysis using Alpha MOS FOX-2000, full-bloomed flowers collected between 9.00 AM to 10.00 AM was used, while for the mobile e-nose, floral volatiles were collected in a polyester bag (Flek-Sampler^®^ Nioibukuro, Omi, Japan) and floral tissues (tepal, pistil and stamen) and developmental stages (green, green-yellow, yellow and full-bloomed) of “Shiny Gold” were weighed to 3 g and analyzed for scent pattern. The polyester bag was filled with O_2_ gas for 60 min and then kept at room temperature. O_2_ gas was used as a control. The length of the acquisition period was one hour. Measurement was taken for 1 min and then washed with O_2_ gas for one minute. We also conducted a postharvest study with cut flowers of “Shiny Gold” and “Yvonne” after 7 days of storage (4 °C). Further, both the sensors were used for the relative scent intensity comparison study in the full-bloomed stage.

### 3.3. Scent Pattern Detection Using E-Nose

The e-nose (Alpha MOS FOX-2000) contained an array of six different metal oxide sensors positioned in a chamber (Alpha MOS, Toulouse, France). The sensors were designated as T30/1, P10/1, P10/2, PA2, P40/1 and T70/2, where *p* and T are the n-type semiconductors. Each sensor detects a particular compound, and the resulting pattern is represented through a radar plot (T30/1 and PA2- organic solvent, P10/1 and P10/2- non-polar solvents, P40/1- fluoride/chloride and T70/2- food flavors and volatile compound) (Alpha MOS, 1998). The analysis was performed by placing a (2 g) flower sample into a 20 mL glass vial with a screwcap. Vials were placed in an auto-sampler (HS-100, CTC Analytics, Toulouse, France). The experiment was conducted in triplicates. Ambient air was used as the control. Analytical conditions were optimized to obtain maximum sensor responses (Table S1). The vials were equilibrated at 40 °C for 5 min. After this stage, the volatile compounds from the headspace moved to an array of sensors at a rate of 150 mL min^−1^. Signal recording was maintained for 120 s and an interval of one second was maintained between two analytical points. After signal processing, the fingerprint map of the e-nose sensor was subjected to dimensionality reduction using principal component analysis (PCA). PCA and radar plots were obtained using Alpha Soft version 12.45.

### 3.4. Collection of Volatiles Using HS-SPME Combined with GC–MS Analysis

*Freesia* “Shiny Gold”, “Yvonne”, “10C3-894” and “10C3-424” were harvested at anthesis and cut inflorescence and were placed immediately in deionized water under laboratory condition at 25 °C. For headspace solid-phase microextraction (HS-SPME), individual flowers were cut from the inflorescence and weighed (2 g). Samples were placed inside a 20 mL glass vial and sealed with polytetrafluoroethylene (PTFE) silicone septum (Agilent Technologies, Inc., Santa Clara, CA, USA). Volatile compounds were adsorbed onto 80 μm divinylbenzene–carboxen–polydimethylsiloxane fiber [DVB/C-WR/PDMS] (PAL SYSTEM, Switzerland) for 10 min at 30 °C. The SPME syringe was injected into the injector port of the GC–MS for desorption.

### 3.5. Analyses of Volatile Compounds Using GC–MS

Volatile compounds were analyzed using GC–MS manufactured by Agilent Technologies, Inc. (model 7890B). A capillary column (Hewlett-Packard-5MS) with 30 m × 0.25 mm and 0.25 μm-thickness was employed. Compounds were extracted in 20 min, and the inlet was operated in splitless mode. The experiment was conducted in triplicates. Analytical conditions are presented in Table S2. GC–MS system was operated with the following temperature program: Oven temperature 40 °C to 3 °C min^−1^, followed by an increase in temperature to 150 °C at a rate of 20 °C min^−1^, then increased to 240 °C and finally temperature was held for 2 min. Helium was used as carrier gas at a flow rate of 1 mL min^−1^. The electron energy was 70 eV. Mass spectra were obtained by automatic scanning at *m*/*z* 20 to 500 amu. Identification of compounds was achieved by comparing the mass spectra with the national institute of standards and technology (NIST 14) library, at a match factor of ≥80, and their retention indices (RIs) were compared with literature values. The data were processed using mass hunter qualitative analysis workflow software (Agilent Technologies Inc., CA, USA). The RIs were calculated relative to a C7-C40 alkane standard (Sigma Aldrich, St.Louis, MO, USA) separated on the HP-5 MS capillary column under the same GC–MS analysis conditions.

### 3.6. RNA Isolation and cDNA Synthesis

Bud (S1) and full-bloomed (S2) flowers were harvested from four cultivars. They were immediately frozen in liquid nitrogen and stored at −80 °C until further use. Total RNA was extracted using a Plant RNeasy^®^ Mini kit, following the manufacturer’s protocol (Qiagen, Germantown, MD, USA). RNA quality was analyzed using a spectrophotometer (Quick Drop, Molecular Devices, San Jose, CA, USA), and integrity was checked with 1% agarose gel electrophoresis in 1x MOPS buffer. Five hundred nanograms of the total RNA was used for synthesizing cDNA. cDNA was synthesized in a final reaction volume of 10 µL from total RNA (0.5 µg) using a Prime Script 1st Strand cDNA Synthesis Kit (Takara, Japan). The resulting product was diluted to a total volume of 100 µL with RNase free water before use in qPCR assays.

### 3.7. Quantitative Real-Time PCR Analysis

qRT-PCR was performed using fluorescent dye SYBR Green (TaKaRa, Shiga, Japan) on a CFX96 real-time PCR detection system (Bio-Rad, Hercules, CA, USA). Primers used in the study were previously reported [16] and are listed in Supplementary Table S3. The qRT-PCR analysis was performed in a final reaction volume of 10 µl, containing a cDNA template, 200 nM each primer and 5 µL 2 × SYBR Premix Ex Taq (TaKaRa, Beijing, China). The experiment was carried out with three biological and technical replications. PCR reaction conditions were as follows: 95 °C for 3 min, followed by 35 cycles of 95 °C for 10 s, 55 °C for 30 s, 72 °C for 30 s and 95 °C for 30 s. The melt curve was checked to ensure the specificity of each primer pair. ASK 1 (F: 5′-CACACCATGCGTCTTCTTGA-3′ and R: 5′ CGCATAGTTTGAGCTGGTGA-3′) was used as the reference gene to calculate the relative fold-difference based on the comparative cycle threshold (2^−∆∆ct^) value [34].

## 4. Conclusions

In conclusion, the identified major VOCs of “Shiny Gold” flower were linalool (38.7%), β-myrcene (3.6%), D-limonene (5%), β-ocimene (29.6%), allo-ocimene (1.3%) and α-terpineol (4.5%). They are all part of the monoterpenoid biosynthesis pathway (Kyoto Encyclopedia of Genes and Genomes database). By integrating volatile profiles with gene expression analysis, we could infer that the variation in the relative proportion of *TPS* genes and volatile compounds may alter the floral scent profile in *Freesia*. However, further studies on the transcription factors governing the site of emission and transcripts involved in scent emission may lead to a better understanding of the molecular mechanism regulating floral scent intensity, and such findings may also be useful in other commercial flower crops.

## Figures and Tables

**Table 1:** Olfactory test of four *Freesia* cultivars; “Shiny Gold”, “Yvonne”, “10C3–894”and ‘10C3–424′.

	Result of Olfactory Test			
	“Shiny Gold”	“Yvonne”	‘10C3-894’	‘10C3-424’
Scent intensity ^a^	Level 4 ^a^	Level 4	Level 5	Level 1

^a^ Level: 1—very weak; 2—weak; 3—medium; 4—strong and 5—very strong.

**Table 2:** Percentage of volatile compounds identified in *Freesia* cultivars using HS-SPME-GC–MS.

SI. No.	Molecular Formula	RT (min)	RI ^a^	Compounds	Relative Content (%) ^b^ ± SD											
					“Shiny Gold”			“Yvonne”			“10C3-894”			“10C3-424”		
				**Monoterpenes**												
1	C_10_H_16_	8.3	925	α-Thujene	0.1	±	0.0 ^c^	-		-	-		-	-		-
2	C_10_H_16_	10.9	989	β-Myrcene	3.6	±	0.0	4.1	±	0.7	5.8	±	0.3	-		-
3	C_10_H_16_	11.9	1013	(+)-4-Carene	0.5	±	0.0	0.6	±	0.1	0.7	±	0.0	-		-
4	C_10_H_16_	12.4	1024	D-Limonene	5.0	±	0.1	-		-	5.2	±	0.3	3.5	±	1.9
5	C_10_H_16_	12.9	1035	2-Norpinene,3,6,6-trimethyl	2.4	±	0.1	2.6	±	0.5	1.5	±	0.1	-		-
6	C_10_H_16_	13.6	1051	β-Ocimene	29.6	±	1.7	27.1	±	3.4	3.4	±	0.5	1.5	±	0.8
7	C_10_H_16_	13.8	Y^′^-Terpinene	1055	0.5	±	0.0	0.5	±	0.2	0.5	±	0.0	-		-
8	C_10_H_16_	15.1	1084	Terpinolene	0.9	±	0.0	1.0	±	0.3	-		-	-		-
9	C_10_H_18_O	16.0	1104	Linalool	38.7	±	2.2	40.9	±	3.9	62.4	±	5.5	23.4	±	13.7
10	C_10_H_14_	16.2	1108	1,3,8-*p*-Menthatriene	0.1	±	0.0	-		-	-		-	-		-
11	C_10_H_16_	17.0	1126	2,4,6-Octatriene, 2,6-dimethyl-, (E,E)-	1.8	±	0.1	3.2	±	1.9	-		-	-		-
12	C_10_H_16_	17.5	1137	allo-Ocimene	1.3	±	0.1	2.1	±	1.3	0.6	±	0.1	-		-
13	C_10_H_18_O	19.8	1187	α-Terpineol	4.5	±	0.5	1.7	±	1.8	4.1	±	0.9	-		-
14	C_10_H_16_O	21.0	1213	β-Cyclocitral	0.4	±	0.2	0.2	±	0.1	-		-	-		-
15	C_10_H_18_O	22.6	1248	Nerol	0.1	±	0.0	-		-	-		-	-		-
				**Sesquiterpenes**												
16	C_15_H_24_	27.8	1367	α-Cubebene	-		-	-		-	2.4	±	0.1	-		-
17	C_15_H_24_	28.8	1390	α-Cyperene	-		-	-		-	0.4	±	0.0	-		-
18	C_13_H_20_O	32.4	1480	trans-β-Ionone	0.1	±	0.1	0.5	±	0.3	-		-	-		-
19	C_15_H_24_	32.7	1487	α-Selinene	-		-	-		-	2.0	±	0.4	-		-
					89.6			84.5			84.1			28.4		

^a^ Retention indices calculated against *n*-alkane C7-C40; ^b^ relative contents (%) = (area under peak/total peak area) × 100; ^c^ all data are presented as mean ± SD (*n* = 3).

## Supplementary Materials

The following are available online at https://www.mdpi.com/2223-7747/9/11/1597/s1, Table S1: E-nose analytical conditions, Table S2. GC-MS analytical conditions, Table S3. Details of the primers are listed in the table, Table S4. Chromatogram of ‘Shiny Gold’, ‘Yvonne’, ‘10C3-894’ and ‘10C3-424’ obtained from GC-MS analyses.
