# Supplementary material for: Analysis of Relative Scent Intensity, Volatile Compounds and Gene Expression in Freesia “Shiny Gold”
Source: Plants (Basel). 2020 Nov 17;9(11):1597. doi: 10.3390/plants9111597 (PMC7698779; doi:10.3390/plants9111597)
Supplement: Supplementary file 1 [file plants-09-01597-s001.pdf]

Supplementary Materials: Table S1. E-nose analytical conditions, **Table S2.** GC-MS analytical conditions, **Table S3.** Details of the primers are listed in the table. **Table S4.** Chromatogram of 'Shiny Gold', 'Yvonne', '10C3-894' and '10C3-424' obtained from GC-MS analyses.

Table S1. E-nose analytical conditions

**Headspace injection**	
Injected volume (μl)	1000
Injected speed (μl/s)	1000
**Acquisition parameters**	
Acquisition time (s)	500
Acquisition period (s)	1
Acquisition duration (s)	120
**Agitator**	
Agitation speed (rpm)	500
**Oven**	
Incubation time (s)	120
Incubation temperature (∘C)	40
Flow (ml/min)	150

Table S2. GC-MS analytical conditions

Incubation temperature	30 ˚C for 10 min
Desorption time	1 min
Injection penetration	50 mm
**SPME**	
Fiber type	Divinylbenzene-carboxen polydimethylsiloxane
Coating	80 μm
**GC**	
Inlet temperature	230 ∘ C
Gas type	Helium
Flow rate	1 ml/min
Oven condition	40 ∘ C
Column	30 m X 0.25 mm, 0.25 μm
Inlet mode	Splitless
Electron Ionization mode	70 eV
Mass scan range	20-500 amu (3.0 scans/sec)

Table S3. Details of the primers are listed in the table

S. No.	Gene ID	Gene name	Forward Primer*	Reverse Primer*
1	101085	*TPS 2*	CTTCAGTGTCTCAGCCTTTG	ACTTCCTCCTTTAACTTCCTA
2	37065	*TPS 3*	CCAGATGTAGTACGCCAGTC	TGCTACCGATTTCAGTGATT
3	37061	*TPS 4*	CCACTACATTGGAGGATGCC	CAGATTTGTCCACCACCTTG
4	62996	*TPS 5*	TCAGTGTCTCAGCCTTTGCG	TGTTCATCACCCATGTAGCC
5	67117	*TPS 6*	AGACGAAAGAAGAAAGAGGT	TACAGTGAGAAGAAGGCAGA
6	38400	*TPS 8*	CTTGGAGCTTCCACTACATT	CACCACCTTGATAATTCCTT
7	-	*LIS*	CGGTGGAAGGAGAAAATCAA	TAGGCATGTATCCGCTGTGA

*Source: Primers used in the study were previously reported in *Freesia hybrida* [16].

Table S4. Chromatogram obtained from the analysis through GC-MS to detect the volatile compounds of (a) 'Shiny Gold', (b) 'Yvonne', (c) '10C3-894', (d) '10C3-424', and (e) Respective compounds detected and their retention time.

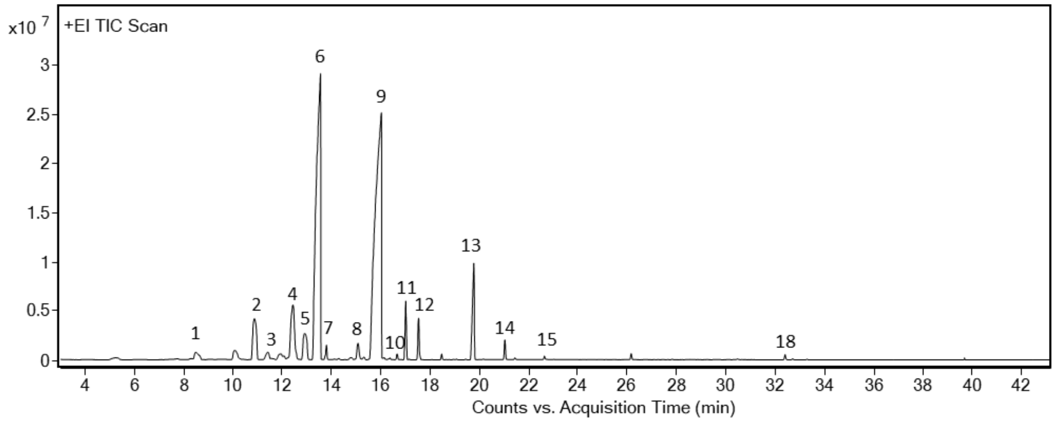

(a)

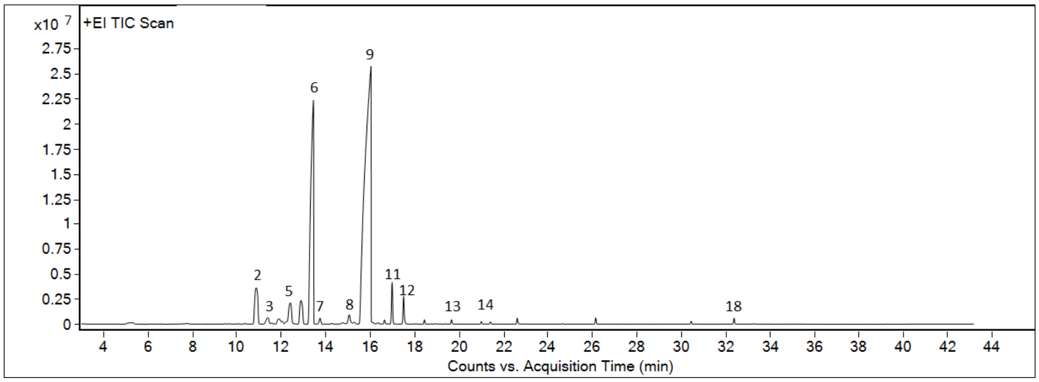

(b)

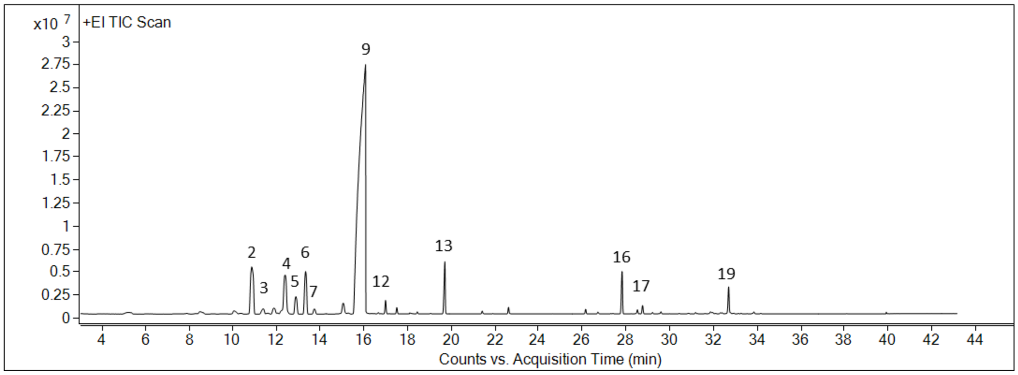

(c)

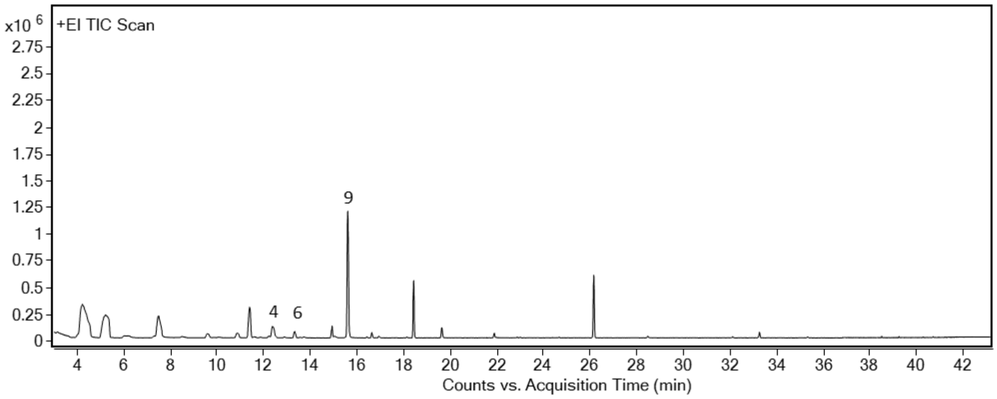

(d)

(e) Respective compounds detected and their retention time

No.	RT (min)	Compounds
1	8.3	α-Thujene
2	10.9	β-Myrcene
3	11.9	(+)-4-Carene
4	12.4	D-Limonene
5	12.9	2-Norpinene,3,6,6-trimethyl
6	13.6	β-Ocimene
7	13.8	ϒ-Terpinene
8	15.1	Terpinolene
9	16.0	Linalool
10	16.2	1,3,8-p-Menthatriene
11	17.0	2,4,6-Octatriene, 2,6-dimethyl-, (E,E)-
12	17.5	allo-Ocimene
13	19.8	α-Terpineol
14	21.0	β-Cyclocitral

15	22.6	Nerol
16	27.8	α-Cubebene
17	28.8	α-Cyperene
18	32.4	trans-β-Ionone
19	32.7	α-Selinene
